# Persistent extreme ultraviolet irradiance in Antarctica despite the ozone recovery onset

**DOI:** 10.1038/s41598-022-05449-8

**Published:** 2022-01-24

**Authors:** Raúl R. Cordero, Sarah Feron, Alessandro Damiani, Alberto Redondas, Jorge Carrasco, Edgardo Sepúlveda, Jose Jorquera, Francisco Fernandoy, Pedro Llanillo, Penny M. Rowe, Gunther Seckmeyer

**Affiliations:** 1 Universidad de Santiago de Chile,, Av. Bernardo O’Higgins, 3363 Santiago, Chile; 2 University of Groningen, Leeuwarden, 8911 CE Netherlands; 3 Center for Environmental Remote Sensing, Chiba University, 1-33 Yayoicho, Inage Ward, Chiba, 263-8522 Japan; 4 State Meteorological Agency (AEMET), Izaña Atmospheric Research Center (IARC), Santa Cruz de Tenerife, Spain; 5 University of Magallanes, Av. Manuel Bulnes 1855, Punta Arenas, Chile; 6 Universidad Andrés Bello, Quillota 980, Viña del Mar, Chile; 7 Alfred Wegener Institute (AWI), Am Handelshafen 12, 27570 Bremerhaven, Germany; 8 NorthWest Research Associates, Redmond, WA USA; 9 Leibniz Universität Hannover, Herrenhauser Strasse 2, Hannover, Germany

**Keywords:** Natural hazards, Atmospheric science

## Abstract

Attributable to the Montreal Protocol, the most successful environmental treaty ever, human-made ozone-depleting substances are declining and the stratospheric Antarctic ozone layer is recovering. However, the Antarctic ozone hole continues to occur every year, with the severity of ozone loss strongly modulated by meteorological conditions. In late November and early December 2020, we measured at the northern tip of the Antarctic Peninsula the highest ultraviolet (UV) irradiances recorded in the Antarctic continent in more than two decades. On Dec. 2nd, the noon-time UV index on King George Island peaked at 14.3, very close to the largest UV index ever recorded in the continent. On Dec. 3rd, the erythemal daily dose at the same site was among the highest on Earth, only comparable to those recorded at high altitude sites in the Atacama Desert, near the Tropic of Capricorn. Here we show that, despite the Antarctic ozone recovery observed in early spring, the conditions that favor these extreme surface UV events persist in late spring, when the biologically effective UV radiation is more consequential. These conditions include long-lasting ozone holes (attributable to the polar vortex dynamics) that often bring ozone-depleted air over the Antarctic Peninsula in late spring. The fact that these conditions have been occurring at about the same frequency during the last two decades explains the persistence of extreme surface UV events in Antarctica.

## Introduction

The stratospheric ozone layer protects life on Earth by absorbing energetic and harmful ultraviolet (UV) radiation^1–4^. The importance of preserving the stratospheric ozone layer has been an international priority since the discovery of ozone depletion^5^, primarily caused by the emission of human-made ozone-depleting substances (ODSs) and the subsequent release of reactive halogen gases in the stratosphere^6^.

Responding to the ozone depletion, the Montreal Protocol banned numerous human-made ODSs^6^. Attributable to the Protocol, the stratospheric halogen concentration resulting from ODSs has been gradually declining^6,7^ and the Antarctic ozone layer recovery has emerged in early spring^7–10^. The Montreal Protocol prevented increases in surface UV radiation at the Earth’s surface^2^, which could otherwise threaten human^11^ and ecosystem^12^ health. The Montreal Protocol has also important co-benefits for climate change mitigation as ODSs are potent greenhouse gases^13,14^ and enhanced UV radiation and climate change may affect the capacity of plants to store carbon through photosynthesis^15^.

Despite the success of the Montreal Protocol, the so-called Antarctic ozone hole continues to occur every spring. The ozone hole results from the severe ozone losses, which reduce the values of total ozone column (TOC) below 220 Dobson Unit (DU)^16,17^, often over an area larger than the Antarctic continent^10^. The ozone hole area is dominated by chemical and dynamical processes that are coupled to the stratospheric temperature. In early spring, the Antarctic ozone depletion is facilitated by the formation of Polar Stratospheric Clouds (PSCs), which provide a surface for heterogeneous chemical reactions involving the ODS-derived halogen gases^6^. In late spring, the Antarctic ozone abundance is controlled by the strength and duration of the polar vortex, which in turn depend on the planetary wave activity^6^.

A cold and strengthened stratospheric polar vortex is associated with large and long-lasting ozone holes, while a warm and disturbed polar vortex is associated with small ozone holes. A metric of the strength and duration of the polar vortex is its breakup date (i.e., the date at which the polar vortex breaks up and the ozone-rich air masses close the ozone hole)^18^. The Antarctic polar vortex strengthened and exhibited a trend toward later breakup dates until the late 1990s^6^. Although it has shown no significant trend during the last two decades, the vortex breakup date has exhibited an enhanced year-to-year variability that appears to be linked to the variability in planetary wave activity^19^. In 2020, a cold and undisturbed polar stratospheric vortex led to a particularly large and long-lasting ozone hole. Conversely, the Antarctic ozone hole was very small in 2019 due to a Sudden Stratospheric Warming (SSW) event that disrupted the polar vortex and allowed unusually strong transport of ozone-rich air into the polar cap^20,21^.

Antarctic ozone depletion has also contributed to disrupt the tropospheric extratropical circulation^22^. From the late 1970s to the early 2000s, the southern annular mode (SAM), the leading mode of natural variability in the Southern Hemisphere extratropical circulation, exhibited a trend toward its positive phase^23,24^. This trend has been partially attributed to Antarctic ozone depletion and resulted from the poleward migration and intensification (especially during austral summers) of the Southern Hemisphere westerly winds^25,26^. Stronger westerly winds (associated with positive trend in the SAM) have caused advection of relatively warm air masses over the Antarctic Peninsula and may also have affected the local cloud cover^27,28^. However, long-term changes in the SAM appear to have paused in the early 2000s due to the stratospheric ozone recovery resulting from the Montreal Protocol^29^.

Severe ozone losses lead to substantial increases in surface UV radiation in Antarctica. During the ozone hole season, the UV index (computed by using the so-called McKinlay and Diffey erythema action spectrum^30^) can be up to 85% higher than estimated pre-ozone hole levels^31^. However, despite the strong influence of ozone levels, surface UV typically reaches its annual maximum in early December, nearly two months after ozone losses peak^31^. The annual maxima of surface UV and ozone losses are unsynchronized mostly due to changes in daily maximum solar elevation. In Polar Regions, the solar elevation at noon varies from winter to summer changing in turn the optical path length of solar radiation through ozone and other absorbers and scatterers; a longer optical path leads to lower irradiance. The influence of the solar elevation explains why, regardless of the atmospheric composition, the surface UV is during winter close to zero over most of the Antarctic continent. The influence of solar elevation also explains why, although the ozone minimum occurs close to the Pole, the highest surface UV in the continent has been measured on the Antarctic Peninsula^31^.

In late November and early December 2020, the highest UV irradiances recorded in the Antarctic continent in more than two decades were measured at the northern tip of the Antarctic Peninsula. Here we show that, despite the Antarctic ozone recovery observed in early spring, the conditions that favor these extreme surface UV events in late spring persist. These conditions include “very large” ozone holes that result in recurrent “very low” TOC values in late spring (close to the summer solstice) over the northernmost part of the continent. Applying a widely used approach for assessing changes in the occurrence probability of extreme events (see “Methods”), we defined “very large” and “very low” values according to percentiles (90th and 10th, respectively) of a daily base climatology (built up by using satellite-derived estimates over the last three decades). As shown below, these metrics are relative to the day of the year in the case of the “very large” ozone holes, and to both the day of the year and the location in the case of the “very low” TOC values. We found that the number of late spring days with “very low” TOC values has not considerably changed since the early 2000s. These findings are consistent with the lack of changes in the frequency of extreme surface UV events observed over the Antarctic Peninsula in the last two decades.

## Results

**Figure 1:**
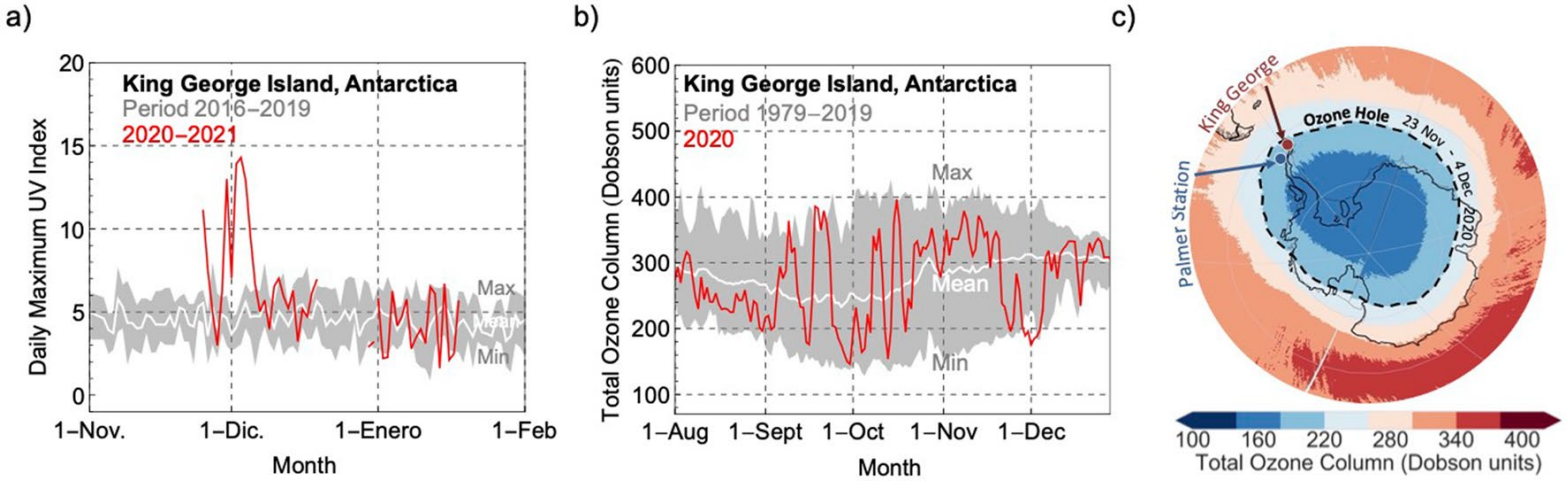
Large ozone losses in late spring 2020 led to extremes of surface ultraviolet radiation at the northern tip of the Antarctic Peninsula. (**a**) Progress of the daily maximum UV index measured on King George Island in late 2020 and early 2021 (red line). The gray shading indicates the highest and lowest values measured over the period 2016–2019 while the white line indicates the mean over the same period. (**b**) Progress of the total ozone column (TOC) for 2020 (red line). The gray shading indicates the highest and lowest values measured over the period 1979–2019 while the white line indicates the mean over the same period. Note that although 2020 was the lowest value for early December, the ozone has shown similarly low values in late spring in the past. Multi-satellite data (TOMS instruments onboard the Nimbus-7 satellite, on the Meteor-3 satellite, and on the Earth Probe satellite, as well as the OMI instrument onboard the Aura satellite) were used for this plot. (**c**) Mean of the total ozone column over Antarctica from 24 November 2020 to 4 December 2020. The dashed line indicates the 220 Dobson units (DU) threshold that defines the ozone hole. The locations of King George Island (62°12′S, 58°58′W) and Palmer Station (64°46′S, 64°03′W) are shown in the plot. Note that the ozone hole was not centered on the pole in late spring 2020. Data from OMI instrument onboard the Aura satellite were used for this plot. The plots were generated using Python’s Matplotlib library^71^.

In late November and early December 2020, we measured extreme values of the surface UV on King George Island (62°12′S, 58°58′W), near the northern tip of the Antarctic Peninsula. Four times within the period Nov. 24th–Dec. 4th, the daily maximum UV index was higher than 11, reaching values more than twice as large as those previously measured at the same location (Fig. 1a). On Dec. 2nd, the noon-time UV index on King George Island peaked at 14.3, the highest value measured in Antarctica in more than two decades and very close to the largest UV index ever recorded in the continent (14.8 at Palmer Station (64°46′S 64°03′W) in early December 1998^32^). Although ground-based measurements of the UV only began in 2016 on King George Island, quality-controlled measurements at Palmer Station, McMurdo station and South Pole are available since 1990^31^. On Dec. 3rd, the erythemal daily dose measured on King George Island (8.1 kJ/m^2^; Fig. S1) was among the highest on Earth, only comparable to those recorded at high altitude sites in the Atacama Desert, near the Tropic of Capricorn^33^.

The extreme surface UV on the Antarctic Peninsula in late November and early December 2020 resulted from the concurrence of several factors:

1. Broken cloud conditions existed. The prevalent cloudy conditions on King George Island attenuate the UV index on average by about 50%^34^. Heavily overcast conditions on the Antarctic Peninsula can reduce the surface UV irradiance by up to 90%^32^. By contrast, in early December 2020, broken clouds allowed extreme UV radiation to reach the surface.
2. The solar elevation was close to its annual maximum (i.e., the summer solstice). In early December, the daily maximum solar elevation is nearly 50° over the Antarctic Peninsula, well above the 20° solar elevation over the South Pole, where, in spite of the deeper ozone losses and the prevalent clear-sky conditions, the UV index has never been higher than 4^35^.
3. The relatively high albedo. Snow-covered surfaces in the interior of the Antarctic continent can enhance the downwelling UV irradiance by up to 50% due to the extremely high albedo (about 95% in the UV part of the spectrum)^36^. Although the albedo is considerably lower at coastal sites on King George Island, where the snow is often thin and patchy in late spring^37^, it may have contributed to enhance the surface UV over the period Nov. 24th–Dec. 4th.
4. The total ozone column was low. TOC values over King George Island (see Fig. 1b) were well below the 220 DU threshold that traditionally defines the ozone hole^16^. In late November and early December 2020, the ozone hole was still deep and large enough to affect the northernmost points of the continent (Figs. 1c and S2, S3). The persistently low TOC values in late 2020 resulted from a cold and undisturbed polar stratospheric vortex. Although it is still under debate, aerosols from the record breaking 2019–2020 Australian wildfire season^38,39^ may have played a role in the 2020 Antarctic ozone hole, proving a surface for heterogeneous chemical reactions. This did appear to have occurred in the case of the volcanic aerosols from the Calbuco eruption in the record-sized 2015 ozone hole^10,40,41^. On December 1st, 2020 the total ozone column diminished to 180 DU, the lowest ever recorded for December over King George Island. Although the ozone is often more depleted in late September (early spring), low solar elevations prevent extreme surface UV even at the northern tip of the Antarctic Peninsula (Fig. S4).

The extreme surface UV measured in late spring 2020 underlines the fact that, although the onset of Antarctic ozone recovery has been observed during early spring^7–10^, low TOC values often persist in late spring when the biologically effective UV radiation is more relevant from an environmental and health perspective.

**Figure 2:**
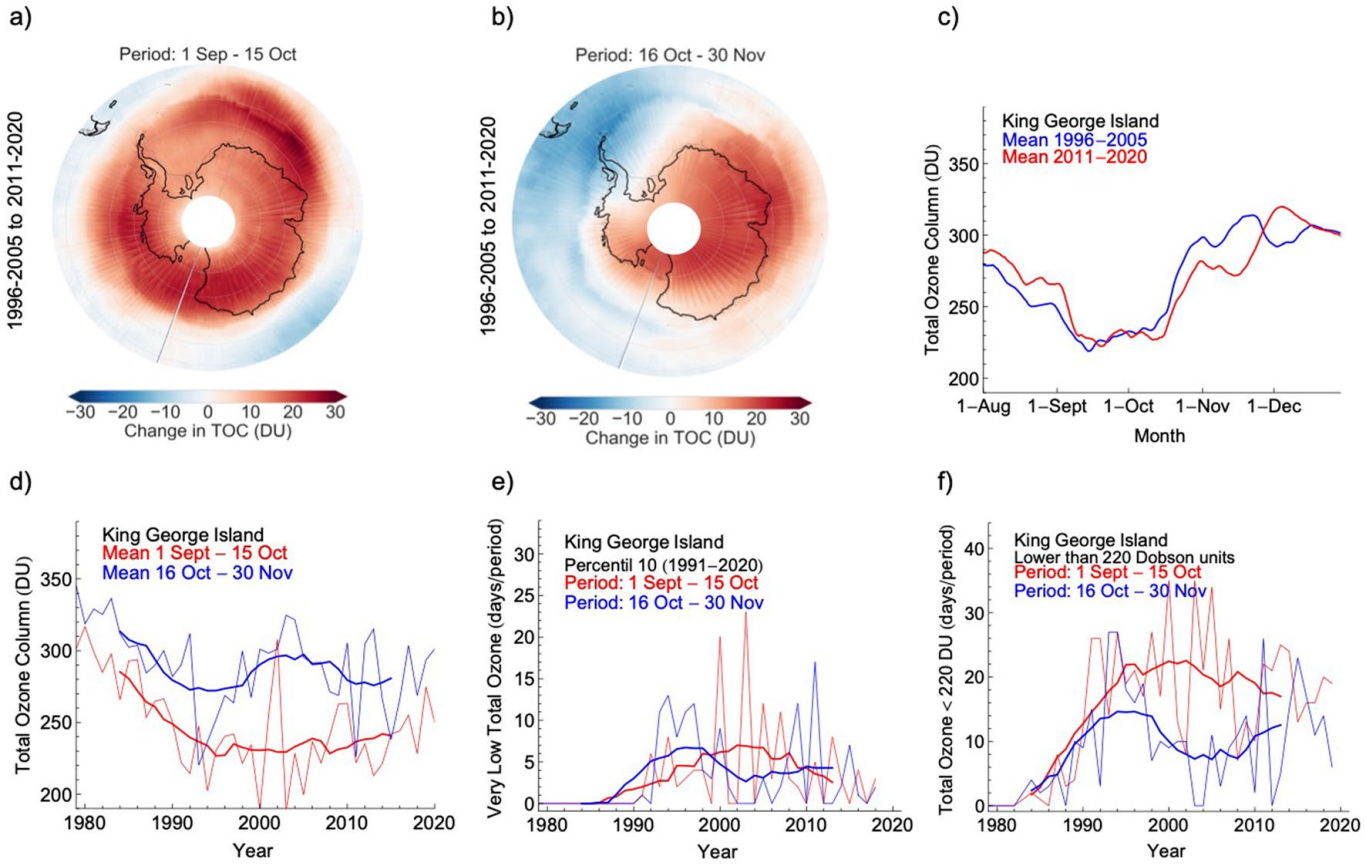
The persistently low values of the total ozone column in late spring favor extremes of UV radiation over the Antarctic periphery. (**a**) Changes from 1996–2005 to 2011–2020 in Antarctic total ozone column (TOC) values averaged from 1 September to 15 October. (**b**) Changes from 1996–2005 to 2011–2020 in Antarctic TOC values averaged from 16 October to 30 November. (**c**) Daily estimates of the TOC values over King George Island averaged over two periods: 1996–2005 (blue line) and 2011–2020 (red line). (**d**) Progress of TOC values averaged from 1 September to 15 October (red line) and from 16 October to 30 November (blue line). (**e**) Number of days with “very low” TOC values (defined according to the 10th percentile) counted over two periods: from 1 September to 15 October (red line) and from 16 October to 30 November (blue line). (**f**) Number of days with TOC values lower than 220 Dobson units (DU) counted over two periods: from 1 September to 15 October (red line) and from 16 October to 30 November (blue line). Bold lines in plots (**d**–**f**) show 11-year centered moving averages. Data from the TOMS instrument on the Earth Probe satellite and from the OMI instrument onboard the Aura satellite were used in plots (**a**,**b**). Data from the Multi Sensor Reanalysis (MSR2) were used in plots (**c**–**f**). The plots were generated using Python’s Matplotlib library^71^.

Despite the year-to-year variability, enhanced by SSW events^20,21^, volcanic eruptions^10,41^ or maybe unprecedented wildfires^38,39^, TOC values exhibit a positive trend. In the last two decades, satellite-derived data show a widespread increase in TOC values averaged from 1 September to 15 October over Antarctica (Figs. 2a and S5a,b). However, TOC values averaged from 16 October to 30 November changed little overall and exhibited opposing regional patterns: while total ozone increased over most of the continent, it slightly declined over most of the Antarctic Peninsula (Figs. 2b and S5c,d). In the case of King George Island, from 1996–2005 to 2011–2020, the total ozone column increased by about 15 DU in August (Fig. 2c) but dropped by about the same amount in November (Fig. 2c). Since the uncertainties associated with satellite data considered in this study are typically about 2%^42,43^, the observed changes of about 15 DU (which is equivalent to about 5%) are likely beyond the uncertainty bounds.

As for the rest of the continent, ozone abundances over King George Island stopped their decline around the ODS peak in 1997. However, TOC trends depend on the time of the year. While the total ozone column averaged from 1 September to 15 October appears to be starting to recover, the trend is less clear in the total ozone column averaged from 16 October to 30 November (Fig. 2d). After an early rapid recovery that ended in the mid-2000s, the 11-year centered moving average of the total ozone for late spring over King George Island has not essentially changed during the last two decades (see bold lines in Fig. 2d).

Although the changes in TOC averages over King George Island range from minor to moderate (Fig. 2c,d), these relatively modest changes mask important changes in the number of days with “very low” TOC values. Here we consider a TOC value to be “very low” if it falls below the 10th percentile of a daily base climatology (built up by using satellite retrievals over the period 1991–2020; see “Methods”). For comparison, we also computed the number of days with TOC values lower than 220 DU.

The number of days with “very low” TOC values over King George Island started to decrease after reaching a maximum about two decades ago, especially for early spring (Fig. 2e). However, for late spring, no considerable changes have been observed in the last decades. Considering the 11-year centered moving average, the number of days with “very low” TOC values declined by half over the period 1 September–15 October (from about 7 days in the early 2000s to about 3 days in the last decade; see Fig. 2e). Over the same period (1 September–15 October), the number of days with TOC values lower than 220 DU declined from about 23 days two decades ago to about 17 days in the last decade (Fig. 2f). However, from 16 October to 30 November, the number of days with “very low” TOC values (and the number of days with TOC values lower than 220 DU) does not show evidence of ongoing decreases after an early rapid recovery that ended in the mid-2000s (Fig. 2e,f).

**Figure 3:**
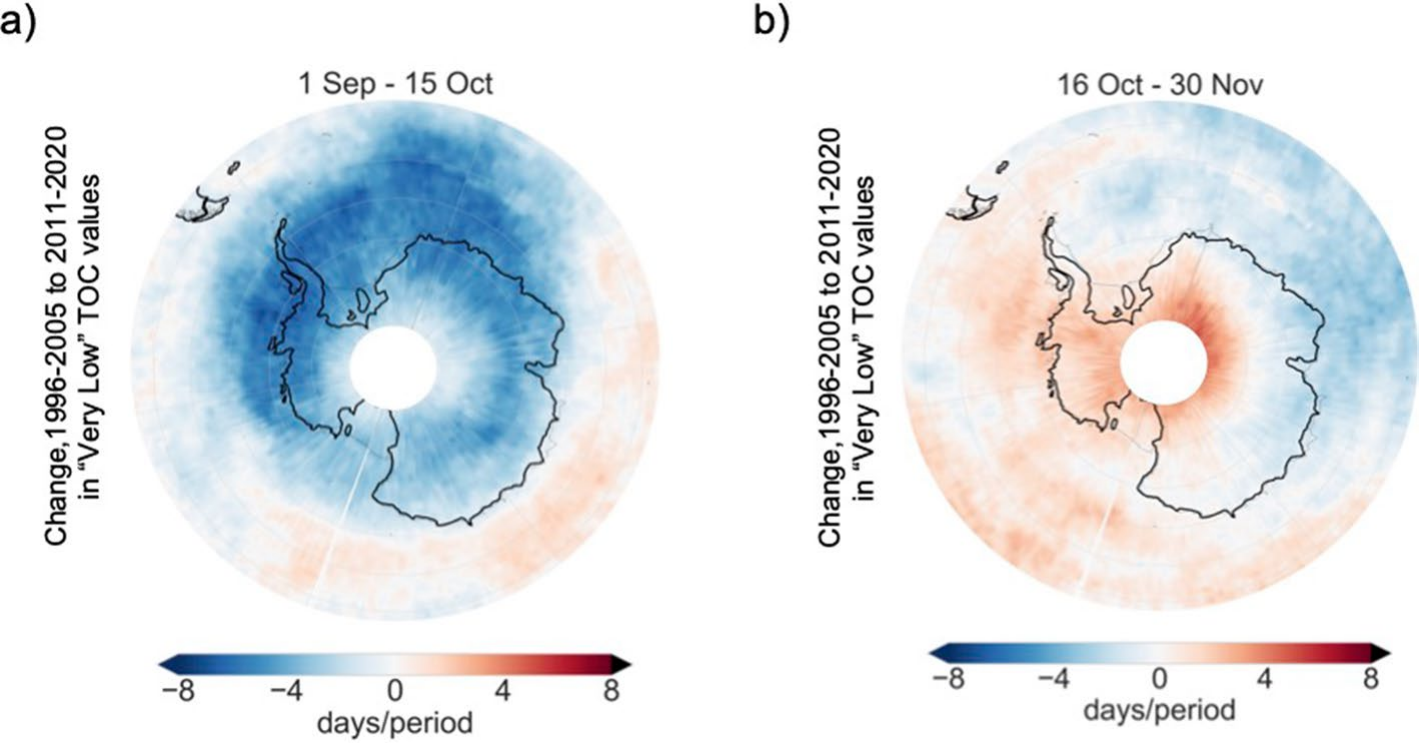
The number of late spring days with “very low” values of the total ozone column has not significantly changed during the last two decades. Changes from 1996–2005 to 2011–2020 in the number of days with “very low” TOC values (defined according to the 10th percentile), over the periods: 1 September–15 October (**a**) and 16 October–30 November (**b**). Data from the TOMS instrument on the Earth Probe satellite and from the OMI instrument onboard the Aura satellite were used in plots (**a**,**b**). The plots were generated using Python’s Matplotlib library^71^.

Similar trends as for King George Island are observed over most of the Antarctic Peninsula, the northern Weddell Sea and the Amundsen Sea. From 1 September to 15 October, the number of days with “very low” TOC values declined from 1996–2005 to 2011–2020 over these regions (Figs. 3a and S6a,b). The changes from 16 October to 30 November are less clear; the number of days with “very low” TOC values did not significantly change for late spring over most of the continent from 1996–2005 to 2011–2020 (Figs. 3b and S6c,d).

The persistence of “very low” TOC values in late spring, when low total ozone amounts coincide with relatively high solar elevation, is particularly consequential for the ~ 400,000 inhabitants of the Southern tip of South America (which includes the Argentinian Santa Cruz and Tierra del Fuego Provinces as well as the Chilean Magallanes Region^44^. As shown in Fig. 3b, the Southern tip of South America is enduring on average about the same number of days with “very low” TOC values in late spring now as two decades ago. Although the ozone column over that region is higher than over the Antarctic Peninsula, the solar elevation is also higher. This is why, the cloudless UV index from satellite estimates is often comparable on both sides of the Drake Passage. This was the case on Dec. 2nd, 2020 (Fig. S7), which prompted the Chilean Weather Service to issue an extreme UV warning for the cities in that region^45^. The possible recurrence of these events promotes the urgent need for ground-based measurements of the UV irradiance at the Southern tip of South America.

**Figure 4:**
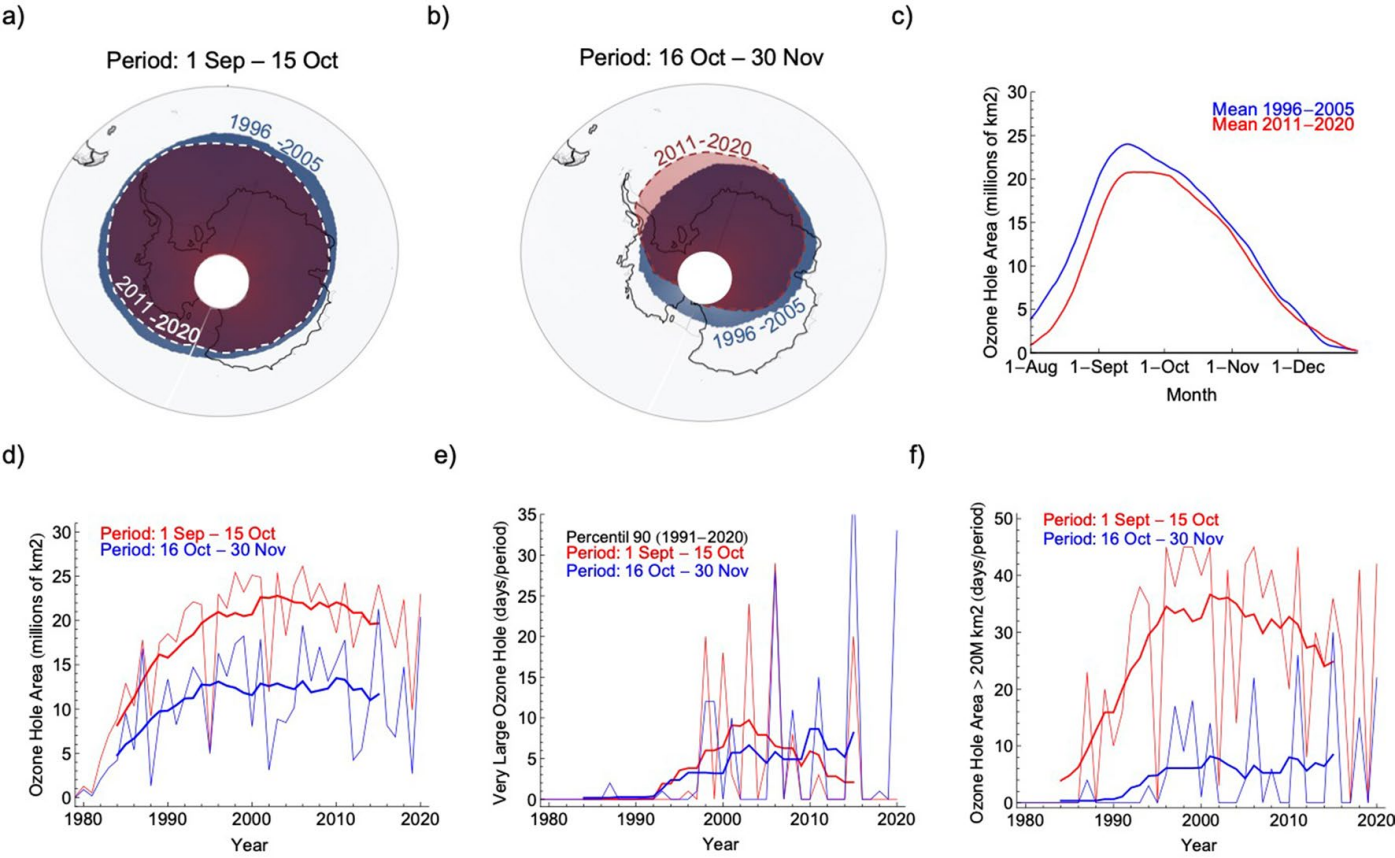
Long-lasting and large ozone holes, that often bring ozone-depleted air over the northern most part of the continent in late spring, have been occurring at about the same frequency during the last two decades. (**a**) Changes from 1996–2005 to 2011–2020 in the ozone hole area averaged from 1 September to 15 October. (**b**) Changes from 1996–2005 to 2011–2020 in the ozone hole area averaged from 16 October to 30 November. (**c**) Daily estimates of the ozone hole area averaged over two periods: 1996–2005 (blue line) and 2011–2020 (red line). (**d**) Progress of ozone hole area averaged from 1 September to 15 October (red line) and from 16 October to 30 November (blue line). (**e**) Number of days with a very large ozone hole area (defined according to the 90th percentile) counted over two periods: from 1 September to 15 October (red line) and from 16 October to 30 November (blue line). (**f**) Number of days with an ozone hole area larger than 20 million km^2^ counted over two periods: from 1 September to 15 October (red line) and from 16 October to 30 November (blue line). Bold lines in plots (**d**–**f**) show 11-year centered moving averages. In plots (**a**,**b**) the ozone hole was estimated considering the area within which, on average over the decades either 1996–2005 or 2011–2020, daily TOC values were lower than 220 Dobson units (DU) on twenty or more days per period. Data from the TOMS instrument on the Earth Probe satellite and from the OMI instrument onboard the Aura satellite were used in plots (**a**,**b**). Data produced by the Laboratory for Atmospheres at NASA's Goddard Space Flight Center were used in plots (**c**–**f**). The plots were generated using Python’s Matplotlib library^71^.

The recurrence of “very low” TOC values results from persistently large and long-lasting ozone holes in late spring. In the last two decades, satellite-derived data show a sizeable reduction in the area of the Antarctic ozone hole averaged from 1 September to 15 October (Fig. 4a). However, the reduction in the area of the ozone hole is less suggestive from 16 October to 30 November (Fig. 4b; despite an eastward shift that has changed the regional distribution of ozone^46^. The seasonality of changes in ozone hole extent is shown in Fig. 4c. From 1996–2005 to 2011–2020, the ozone hole area dropped by about 5 million km^2^ in August and in September but did not change meaningfully in November and December.

The ozone hole area depends on dynamical and chemical processes that are coupled to the stratospheric temperature. Roughly mirroring changes in stratospheric temperature (Fig. S8), the ozone hole area increased from the early 1980s until reaching its peak over Antarctica about two decades ago (Fig. 4d). While the ozone hole area averaged from 1 September to 15 October appears to be steadily declining from the early 2000s, there is no apparent change in the ozone hole area averaged from 16 October to 30 November. The 11-year centered moving average of the ozone hole area for late spring has been stable during the last two decades (Fig. 4d; see bold blue line).

The number of days with a “very large” ozone hole exhibits similar trends. Here we consider an ozone hole to be “very large” if its area falls above the 90th percentile of a daily base climatology (built up by using satellite-derived estimates over the last three decades; see “Methods”). For comparison, we also computed the number of days with an ozone hole area larger than 20 million km^2^.

In early spring (1 September to 15 October), when Antarctic ozone depletion is dominated by chemistry, the days with a “very large” ozone hole have fallen to almost zero since reaching a maximum in the early 2000s (Fig. 4e; see bold red line). Over the same period, the days with an ozone hole area larger than 20 million km^2^ declined from about 40 days in the early 2000s to about 25 days in 2020 (Fig. 4f; bold red line). The fact that the 11-year centered moving average in Fig. 4e (see bold red line) is rapidly approaching zero is why the Montreal Protocol has been hailed as the most successful environmental treaty ever.

However, the ozone hole area has seen little progress in late spring, when the ozone abundance is primarily controlled by transport rather than chemistry. From 16 October to 30 November, the number of days with a “very large” ozone hole (and the days with an ozone hole area larger than 20 million km^2^) has not considerably changed during the last two decades (see blue line in Fig. 4e,f).

The persistence of “very large” ozone holes in late spring is consistent with the lack of statistically significant trends in the surface UV measured on the Antarctic Peninsula during the ozone hole season. Although ground-based measurements at Palmer Station exhibited a drop in the late 1990s, they have shown no changes on average afterwards (Fig. S9); the average erythemal irradiance for October and November is still estimated to be about 30–60% higher than the estimate for the years 1963–1980 pre-ozone hole levels^31^.

## Discussion

The extreme surface UV measured on the Antarctic Peninsula in late 2020 underlines the fact that, although the Antarctic ozone is recovering in early spring, low TOC values persist in late spring. For early spring, when the Antarctic ozone depletion is dominated by chemistry, the number of days with “very low” TOC values appears to be steadily recovering. However, the trend is less clear for late spring, when the ozone abundance is controlled by polar vortex dynamics.

The recurrence of “very low” TOC values over Antarctica results from persistently large ozone holes in late spring, when the Antarctic ozone abundance depends on the characteristics of the stratospheric polar vortex, such as its strength and duration. Although the vortex breakup date has shown no significant trend during the last two decades^6^, it has exhibited an enhanced year-to-year variability during the last two decades that appears to be linked to variability in planetary wave activity.

Our understanding of the processes that determine the interannual variability of the planetary wave activity is still incomplete. However, recent efforts have shown that anomalously large November ozone hole events are related to planetary wave divergence in the stratosphere between 60° and 90°S, which has in turn been partially attributed to less upward propagation of planetary waves from the troposphere^47^. In addition, triggered by planetary waves, weakening polar-vortex events appear to have become more frequent since the 2000s^19^. This is particularly relevant for the Antarctic Peninsula and associated archipelagos as variations in ozone in this region are primarily controlled by transport rather than chemistry. Weakening polar-vortex events may enable ozone-depleted polar air to move into lower latitudes, leading to extreme surface UV events.

While ODS concentrations drove ozone depletion in recent decades, the ozone recovery progress in upcoming decades will also be determined by the concentration of greenhouse gases (GHG)^6^. By cooling the stratosphere, increasing GHG concentrations change the rate of heterogeneous reactions, thus affecting ozone abundances. The rise in GHG concentrations projected for the next decades may offset part of the stratospheric warming caused by increasing ozone abundance, favoring in turn a strengthened polar vortex^6^.

As the ozone layer continues to recover, the influence of the Antarctic ozone hole on the tropospheric circulation will weaken, increasing the relative importance of the rising concentrations of GHG^48^. A robust positive trend in the SAM is projected under high-emission scenarios^49^, which may affect the cloud cover and in turn the surface UV on the Antarctic Peninsula. Long-term trends in the SAM could, by changing cloud patterns, induce trends in the surface UV on the Antarctic Peninsula. However, the SAM has seen no significant change since the early 2000s^29^, which is consistent with the lack of surface UV trends observed during the last two decades at Palmer Station^31^. The GHG emission trajectory will determine if the poleward migration and intensification of the westerly winds resume^50^ and if further long-term cloudiness changes occur in the region.

Although we found that the days with a “very large” ozone hole are nearly over during early spring, changes in the ozone hole area during late spring have been minor, and persistently long-lasting and large ozone holes remain possible. The frequent “very large” ozone holes in late spring enable extreme surface UV events over the Antarctic Peninsula (and also over the Southern tip of South America).

## Methods

### Ground-based measurements

Measurements of the surface UV spectra on King George Island (since 2016) and at Palmer Station (since 1990) are carried out by using spectroradiometer systems that fulfill the specifications defined by the World Meteorological Organization (WMO)^51^ and the Network for the Detection of Atmospheric Composition Change (NDACC)^52^. The systems are based on monochromators, a Bentham DMC150 in the case of King George Island and a Biospherical SUV-100 in the case of Palmer Station. These instruments produce measurements with uncertainties of up to 10% for UV-B wavelengths (290–315 nm) and up to 4% for UV-A wavelengths (315–400 nm)^53^. Measurements at Palmer Station are available at: https://www.ndaccdemo.org.

The UV-A and UV-B irradiance were computed from the spectral measurements by calculating the integral within the ranges 315–400 nm and 290–315 nm, respectively. The erythemal irradiance was computed from the spectral Measurements by calculating the integral of the spectral UV irradiance in the range 250–400 nm, weighted by using the so-called McKinlay-Diffey Erythema action spectrum^30^. The dimensionless UV Index was obtained by scaling with 40 m^2^/W the erythemal irradiance; the World Health Organization considers that values of the UV index greater than 11 stand for extreme risk of harm from unprotected sun exposure^54^.

Additional comparisons (Fig. S4) involved UV estimates rendered by the UVSPEC radiative transfer model^55^. In the UV spectral range, this model has been validated by systematic comparisons with ground-based measurements under cloudless conditions in other geographic regions^56,57^. The model used the DIScrete Ordinates Radiative Transfer (DISORT) solver^58^ and the extraterrestrial spectrum by Gueymard^59^.

### Satellite data

Ozone data for 1979–1992 are from the Total Ozone Mapping Spectrometer (TOMS) instrument on the Nimbus-7 satellite. The data for 1993–1994 are from the TOMS instrument on the Meteor-3 satellite. The data for 1996–2004 are from the TOMS instrument on the Earth Probe (EP-TOMS) satellite. The data starting from 2005 are from the Ozone Monitoring Instrument (OMI) onboard the Aura satellite. For further analysis, all datasets were regridded to a common resolution: 1.0 × 1.25°. The datasets are available at https://disc.gsfc.nasa.gov/datasets/. Validation efforts of these products has been primarily based on comparison with a network of Dobson and Brewer ground stations. For example, EP-TOMS total column ozone averages agree within ± 1% with the station average at latitudes higher than 50ºS while OMI-TOMS agrees with a network of 76 stations with an uncertainty of only 0.6%^60^. The uncertainties associated with satellite data are generally estimated based on a comparison of data from different instruments. These efforts have shown differences between the satellite instruments considered in this study are typically within ± 2%^43^.

For the ozone hole area, we used the data produced by the Laboratory for Atmospheres at NASA's Goddard Space Flight Center. In this case, to calculate the ozone hole area missing areas (bad orbits and polar night) are filled using assimilated ozone data (from the Modern-Era Retrospective analysis for Research and Applications (MERRA)^61^ for 1979 through June 2016, MERRA-2 for July 2016 through August 2017^62^, and GEOS FP from September 2017 on) produced by the Goddard Earth Observing System Data Assimilation System (GEOS DAS). MERRA and MERRA-2 use a version of the GEOS model with the Gridpoint Statistical Interpolation (GSI) atmospheric analysis developed jointly with NOAA/NCEP/EMC. The datasets are available at: https://ozonewatch.gsfc.nasa.gov/meteorology/SH.html.

For specific locations (King George Island, for example), we also used the ozone data for 1979–2018 from TM3DAM version 4.4 Overpass Multi Sensor Reanalysis (MSR2)^63^. The datasets are available at https://www.temis.nl/protocols/o3field/overpass_msr2.php. The Observation-minus-Analysis (OmA) statistics show that the bias of the Multi Sensor Reanalysis is less than 1%^42^.

Additional analysis (Fig. S2) involved ozone profiles retrieved from the Limb Profiler of the Ozone Mapping and Profiler Suite (OMPS-LP), aboard the Suomi NPP satellite^64^. Satellite-derived ozone profiles were obtained from the NASA Goddard Space Flight Center web site (Aura Validation data center): https://gs614-avdc1-pz.gsfc.nasa.gov/pub/data/satellite/Suomi_NPP/L2OVP/LP-L2-O3-DAILY/. Prior efforts have shown that, relative to balloon‐borne measurements, the mean bias error of OMPS‐derived Antarctic ozone profiles is generally lower than 0.3 ppmv, regardless of the season^65^.

In the case, of satellite-retrieved estimates (under cloudless conditions) of both UV index and the erythemal daily dose we used data from the Tropospheric Emission Monitoring Internet Service (TEMIS) UV index and UV dose operational data^66^: https://www.temis.nl/uvradiation/UVarchive/uvncfiles.php?Year=2021.

Finally, the minimum Antarctic temperature (Fig. S8) is determined for latitudes south of 50°S by the Laboratory for Atmospheres at NASA's Goddard Space Flight Center. In this case, data are retrieved from the Modern-Era Retrospective analysis for Research and Applications, Version 2 (MERRA-2) assimilation^61^, produced by the Goddard Earth Observing System Data Assimilation System (GEOS DAS). The datasets are available at: https://ozonewatch.gsfc.nasa.gov/meteorology/temp_2021_MERRA2_SH.html.

### Analysis of extremes

We applied a widely used methodology for assessing changes in the occurrence probability of extreme events^67–70^. Over a base period of 30 years (1991–2020), we used a 15-day rolling window of the daily estimate of either the total ozone column (TOC) or the ozone hole area (OHA) in order to form datasets of 450 values for each day. For each day (and also for each grid point in the case of the TOC), the dataset mean defined a daily base climatology from which daily anomalies (either for TOC or for OHA) were in turn calculated. The histograms or the corresponding probability density functions (PDF) of the daily anomalies (the departure of daily estimate from the daily base climatology) allowed us to compute:

- The number of days with “very low” TOC values: the number of days below the 10th percentile of the TOC anomaly distribution corresponding to the base period.
- The number of days with “very large” OHA values: the number of days above the 90th percentile of the OHA anomaly distribution corresponding to the base period.

For each year (from 1979 to 2020), these two metrics were computed over two periods (1 September–15 October and 16 October–30 November).

## Supplementary Information

Supplementary Figures.

## Data Availability

Multi-satellite observations of the total ozone columns are available at https://disc.gsfc.nasa.gov/datasets/. Multi Sensor Reanalysis datasets are available at https://www.temis.nl/protocols/o3field/overpass_msr2.php. Estimates of ozone hole area and the minimum Antarctic temperature are available from the NASA Goddard Space Flight Center web site (Aura Validation data center): https://ozonewatch.gsfc.nasa.gov/meteorology/SH.html and https://ozonewatch.gsfc.nasa.gov/meteorology/temp_2021_MERRA2_SH.html, respectively. Ozone profiles were obtained from the NASA Goddard Space Flight Center web site (Aura Validation data center): https://gs614-avdc1-pz.gsfc.nasa.gov/pub/data/satellite/Suomi_NPP/L2OVP/LP-L2-O3-DAILY/. Satellite-retrieved estimates of both UV index and the erythemal daily dose are available at: https://www.temis.nl/uvradiation/UVarchive/uvncfiles.php?Year=2021. Ground-based UV measurements at Palmer Station are available at: https://www.ndaccdemo.org. Ground-based UV measurements on King George Island and codes are available from the corresponding author on request. All data needed to evaluate the conclusions in the paper are present in the paper and/or the Supplementary Materials.
