# Supplementary material for: Persistent extreme ultraviolet irradiance in Antarctica despite the ozone recovery onset
Source: Sci Rep. 2022 Jan 24;12:1266. doi: 10.1038/s41598-022-05449-8 (PMC8786956; doi:10.1038/s41598-022-05449-8)
Supplement: Supplementary file 1 — Supplementary Figures. [file 41598_2022_5449_MOESM1_ESM.pdf]

Supplementary Information

Persistent Extreme Ultraviolet Irradiance in Antarctica despite the Ozone Recovery Onset.

Raúl R. Cordero[1,*], Sarah Feron[1,2], Alessandro Damiani[3], Alberto Redondas[4], Jorge Carrasco[5], Edgardo Sepúlveda[1], Jose Jorquera[1], Francisco Fernandoy[6], Pedro Llanillo[7], Penny M. Rowe[1,8], Gunther Seckmeyer[9]

1 Universidad de Santiago de Chile. Av. Bernardo O'Higgins 3363, Santiago, Chile.
2 University of Groningen, Leeuwarden 8911 CE, Netherlands.
3 Center for Environmental Remote Sensing, Chiba University, 1-33 Yayoicho, Inage Ward, Chiba, 263-8522, Japan
4 Izaña Atmospheric Research Center (IARC), State Meteorological Agency (AEMET), Santa Cruz de Tenerife, Spain
5 University of Magallanes, Av. Manuel Bulnes 1855, Punta Arenas, Chile.
6 Universidad Andrés Bello, Quillota 980, Viña del Mar, Chile
7 Alfred Wegener Institute (AWI), Am Handelshafen 12, 27570 Bremerhaven, Germany
8 NorthWest Research Associates, Redmond, WA, USA.
9 Leibniz Universität Hannover, Herrenhauser Strasse 2, Hannover, Germany.

* Corresponding Author
Raúl R. Cordero, raul.cordero@usach.cl
Av. Bernardo O'Higgins 3363, Santiago, Chile
+56997352030

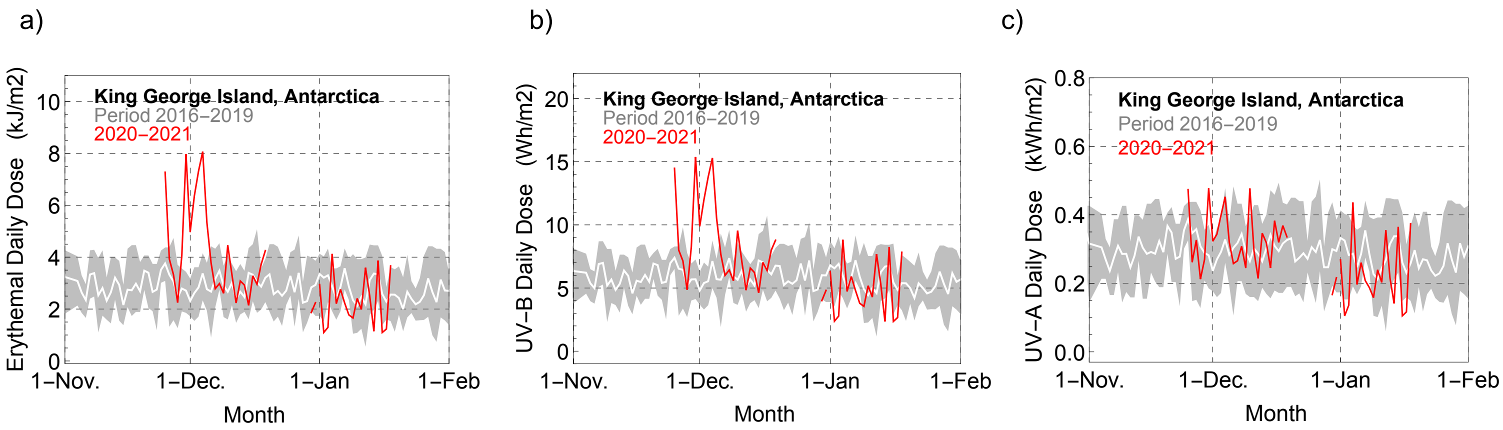

Fig. S1.

The red lines indicate the progress of the daily doses computed from ground-based spectral measurements on King George Island in late 2020 and early 2021. The gray shadings in the plots indicate the highest and lowest values measured over the period 2016-2019, while the white line indicates the mean over the same period.

a) Erythemal daily dose;
b) UV-B daily dose (290-315 nm);
c) UV-A daily dose (315-400 nm).

Ground-based UV measurements on King George Island are available from the corresponding author. The plots were generated using Python's Matplotlib library[71].

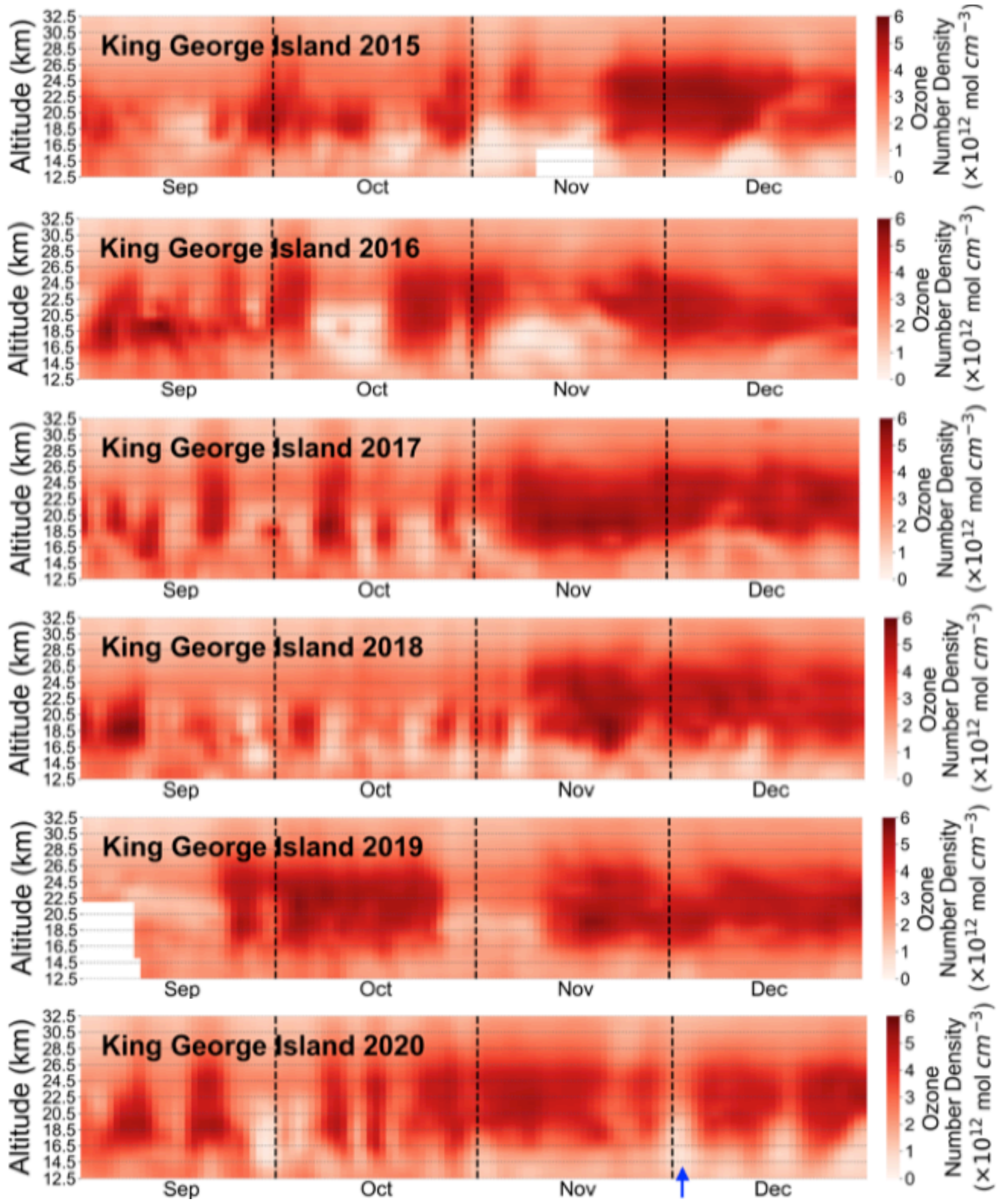

Fig. S2.

Heatmap of the ozone profiles over King George Island form September to December over the period 2015-2020. The blue arrow indicates the ozone losses that led to the extreme surface UV doses shown in Fig. S1. The profiles were retrieved from the Limb Profiler of the Ozone Mapping and Profiler Suite (OMPS-LP), aboard the Suomi NPP satellite. The plot was generated using Python's Matplotlib library[71].

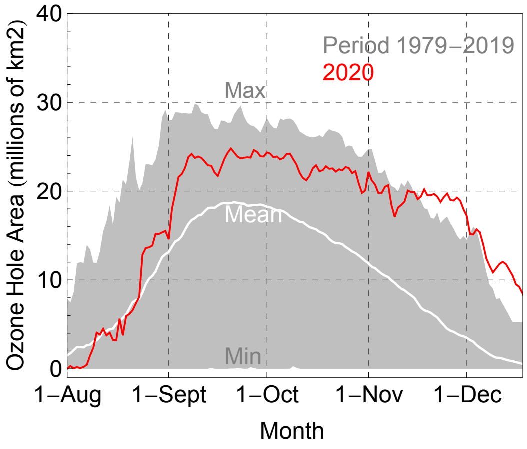

Fig. S3.

The red line indicates the progress of the ozone hole area for 2020. The gray shading indicates the highest and lowest values recorded over the period 1979-2019, while the white line indicates the mean over the same period. Data produced by the Laboratory for Atmospheres at NASA's Goddard Space Flight Center were used in this plot. The plot was generated using Python's Matplotlib library[71].

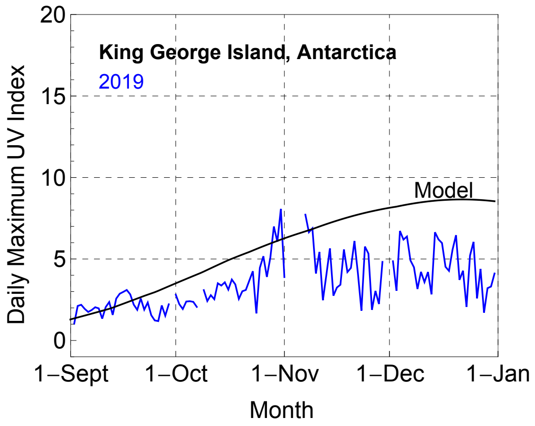

Fig. S4.

The blue line indicates the progress of the daily maximum UV index computed from ground-based spectral measurements on King George Island from September to December 2019. For comparison, the black line indicates the progress of the noontime UV index computed by using the UVSPEC radiative transfer model assuming cloudless conditions and a constant total ozone column (the mean for October computed over the period 1979-2019: 257 DU). Although the model simulation suggests that the highest values of the UV index may occur in December (close to the solstice), this rarely occurs because, as shown in Fig. 1c, the TOC values over King George Island are generally higher in December than in November. The effect of the ozone hole is apparent in early September and early November. The plot was generated using Python's Matplotlib library[71].

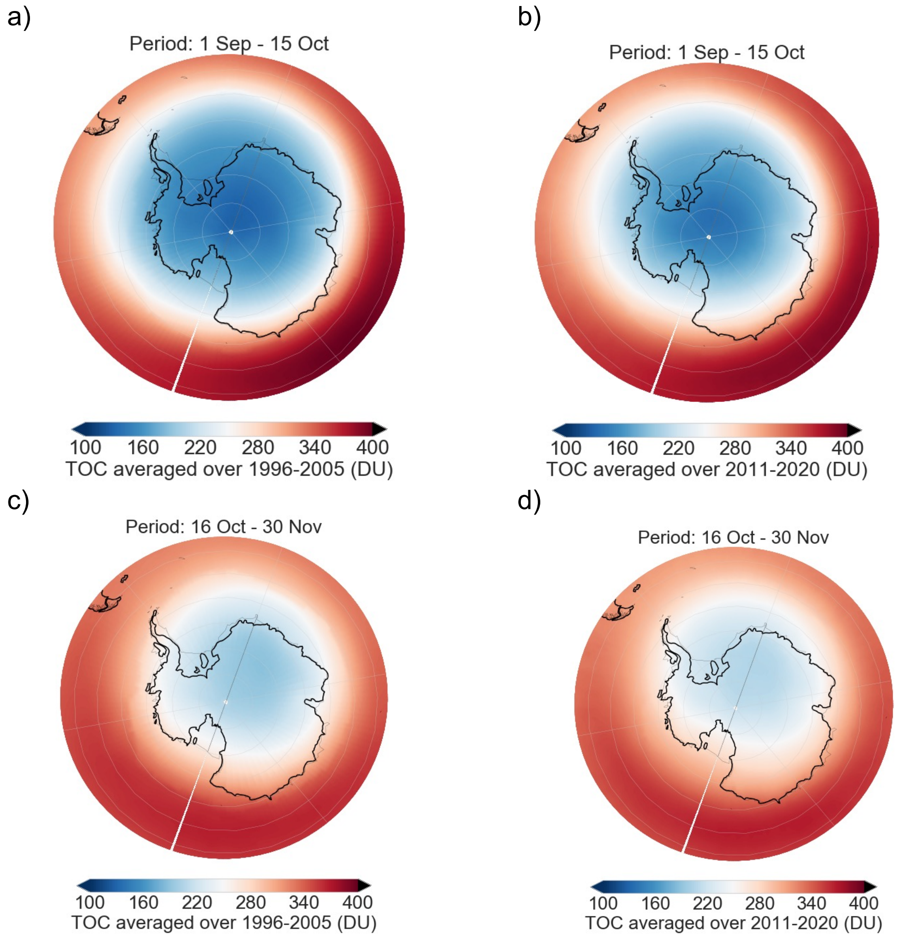

Fig. S5.

First row: Total ozone column (TOC) values averaged from 1 September to 15 October over the period 1996-2005 (a) and over the period 2011-2020 (b).

Second row: TOC values averaged from 16 October to 30 November over the period 1996-2005 (c) and over the period 2011-2020 (d).

Data from the TOMS instrument on the Earth Probe satellite and from the OMI instrument onboard the Aura satellite were used in the plots. The plots were generated using Python's Matplotlib library[71].

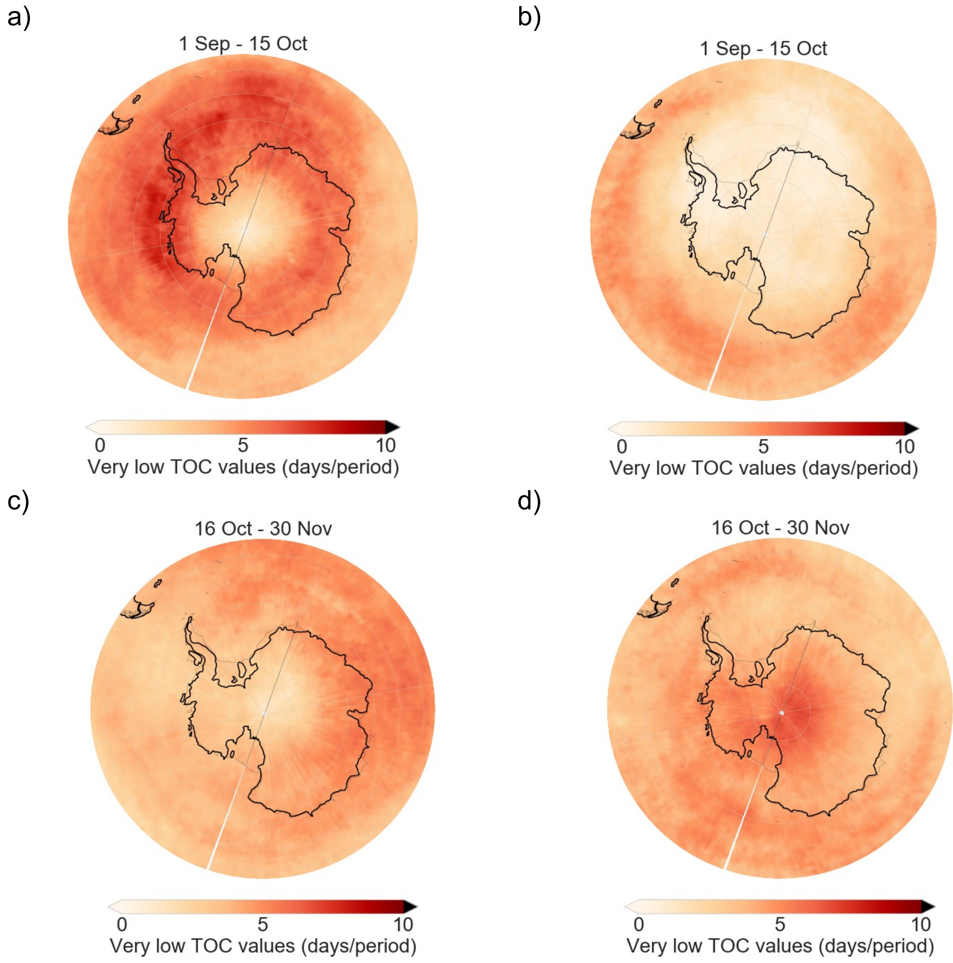

Fig. S6.

First row: Number of days with “very low” TOC values (defined according to the 10th percentile) counted from 1 September to 15 October and averaged over the periods 1996-2005 (a) and 2011-2020 (b).

Second row: Number of days with “very low” TOC values (defined according to the 10th percentile) counted from 16 October - 30 November and averaged over the periods 1996-2005 (c) and 2011-2020 (d).

Data from the TOMS instrument on the Earth Probe satellite and from the OMI instrument onboard the Aura satellite were used in the plots. The plots were generated using Python’s Matplotlib library[71].

a) b)

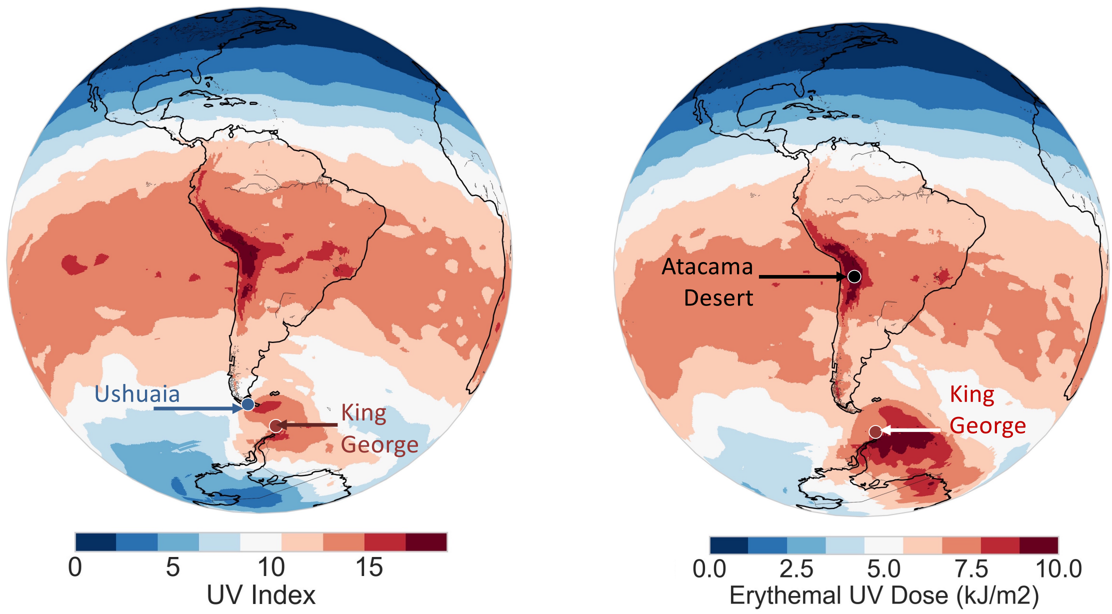

Fig. S7. Satellite-retrieved estimates of the:

a) Clear-sky UV index on Dec. 2nd, 2020. Note that, according to satellite estimates, the UV index was comparable on both sides of the Drake Passage.

b) Clear-sky erythemal daily dose on Dec. 3rd, 2020. Note that, according to satellite estimates, the erythemal daily dose was at the northern tip of the Antarctic Peninsula among the highest on Earth, only comparable to those recorded at high altitude sites in the Atacama Desert, near the Tropic of Capricorn.

Data from the TEMIS UV index and UV dose operational data products were used in the plots. The plots were generated using Python's Matplotlib library[71].

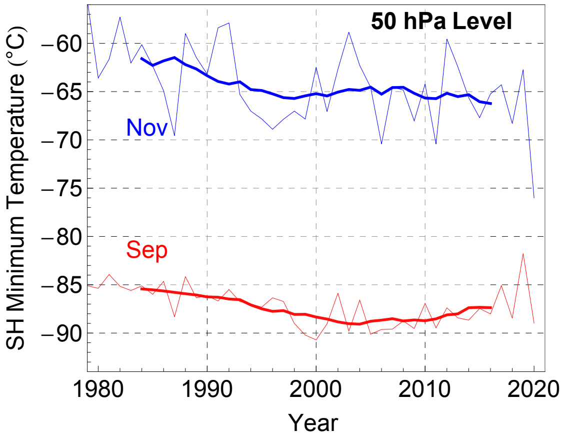

Fig. S8.

Monthly means of 50 hPa Southern Hemisphere (SH) minimum temperature for November (blue) and for September (red). **Bold lines show 11-year centered moving averages**. Data produced by the Laboratory for Atmospheres at NASA's Goddard Space Flight Center were used in this plot. The plot was generated using Python's Matplotlib library[71].

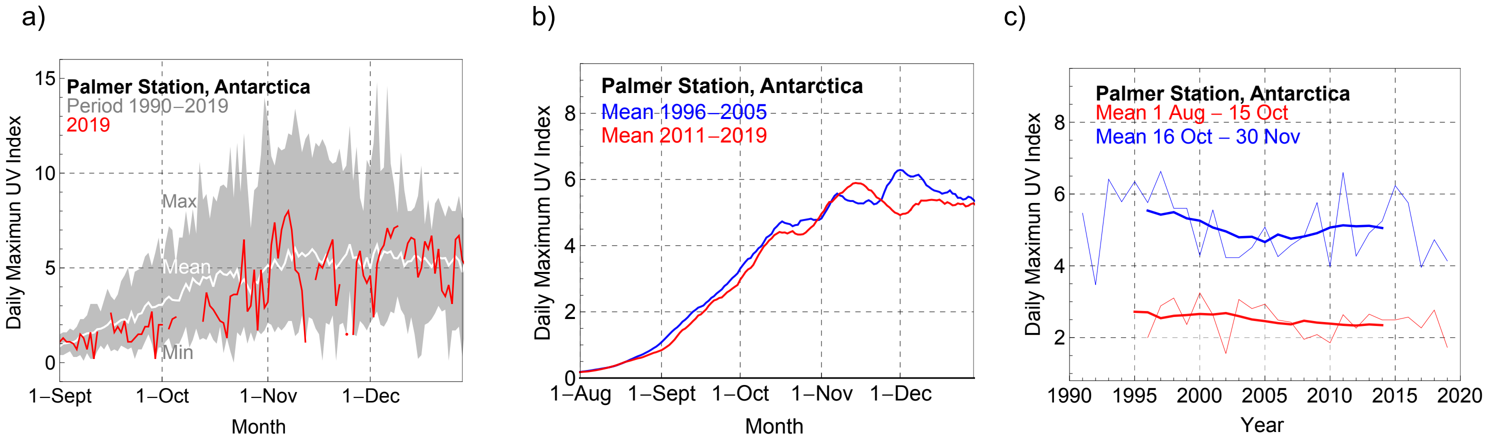

Fig. S9.

a) Progress of the daily maximum UV index measured at Palmer Station in late 2019 (red line). The gray shading indicates the highest and lowest values measured over the period 1990-2019, while the white line indicates the mean over the same period.

b) Daily maximum UV index averaged over two periods: 1996-2005 (blue line) and 2011-2019 (red line).

c) Progress of daily maximum UV index averaged from 1 September to 15 October (red line) and from 16 October to 30 November (blue line).

Bold lines in plot (c) show 11-year centered moving averages. Measurements at Palmer Station are available at https://www.ndaccdemo.org. The plots were generated using Python's Matplotlib library[71].
